# The Life of Pasteur

**Published:** 1902-03

**Authors:** 


### Reviews of Books.

**The Life of Pasteur.** By RENÉ VALLERY-RADOT. Translated from the French by Mrs. R. L. Devonshire. Two volumes. Vol. I. Pp. vii., 293. Vol. II. Pp. vii., 336. Westminster: Archibald Constable & Co. 1902.

The lives of Paget and of Pasteur form instructive volumes to the student of the last century. Lady Priestley has contributed an interesting review of them to the current number of the *Nineteenth Century and After*. We are now only concerned with the latter; and the first of these volumes takes us back to the year 1822, when Louis Pasteur was born at Dôle. There do not appear to be any wonderful legends with which to decorate the story of his early life; but "never was home-sickness more acute" than when, in Paris, he remarked: "If I could only get a whiff of the tannery yard, I feel I should be cured." His early work in lactic, tartaric, and alcoholic fermentation was followed a few years later by his experiments which led to the overthrow of the theory of spontaneous generation.

The endless theories and fancies of forty or more years ago were doomed to destruction by his work on atmospheric dust; for after a year's study, Pasteur reached this conclusion: "Gases, fluids, electricity, magnetism, ozone, things known or things occult, there is nothing in the air that is conditional to life, except the germs that it carries." By this work he laid the basis of microbian pathology as we know it to-day, and he himself "had glimpses of another world beyond the phenomena of fermentation — the world of virus ferments." He often regretted that he was not a medical man, fancying that it might have facilitated his task if he had been armed with diplomas: but in 1874 he obtained the active collaboration of Joseph Lister, who wrote as follows: "Allow me to take this opportunity to tender you my most cordial thanks for having, by your brilliant researches, demonstrated to me the truth of the germ theory of putrefaction, and thus furnished me with the principle upon which alone the antiseptic system can be carried out."

After this, in 1877, came the protracted conflict with Dr. Bastian, to whom he wrote as follows: "Do you know why I desire so much to fight and conquer you? It is because you are one of the principal adepts of a medical doctrine which I believe to be fatal to progress in the art of healing—the doctrine of the spontaneity of all diseases." It has not yet been forgotten how keenly this conflict was fought out, and how completely Pasteur became the victor.

Soon afterwards the subject of splenic fever, of which the microbe had just been discovered by Robert Koch, attracted his attention, and then the chicken cholera, from both of which diseases he was able to produce an attenuated virus which might be used as a prophylactic vaccine.

The reception of Pasteur at the International Medical Congress in London in 1881, and his meeting of the Prince of Wales and the Crown Prince of Germany, form a well told story. "When Mr. Pasteur spoke, when his name was mentioned, a thunder of applause rose from all benches, from all nations. An indefatigable worker, a sagacious seeker, a precise and brilliant experimentalist, an implacable logician, and an enthusiastic apostle, he has produced an invincible effect on every mind."

Such applause as Pasteur continued to receive might well have caused him to be satisfied with his labours in the cause of science; but although proud of his discoveries, they but served as a stimulus to future efforts. If people spoke to Pasteur of the danger of infection, "What does it matter?" he said. "Life in the midst of danger is *the* life, the real life, the life of sacrifice, of examples, of fruitfulness."

It will be remembered that the later years of Pasteur's active life were devoted mainly to the Hydrophobia Problem and the establishment of the Pasteur Institute. There his character revealed itself on every occasion. "Every morning, with a step rendered heavy by age and ill-health, he went from his rooms to the Hydrophobia Clinic, arriving there long before the patients. He superintended the preparation of the vaccinal marrows; no detail escaped him. When the time came for inoculations, he was already informed of each patient's name, sometimes of his poor circumstances, and he had a kind word for everyone, often substantial help for the very poor."

Few men live to see so full a display of the fruit of their labours; but no one realised better than Pasteur, shortly before his death in 1895, that "There is still a great deal to do."

The translator has accomplished her task so well that the writing appears to be original, and the publisher has produced two volumes of which the actual weight is in inverse proportion to the gravity and value of the contents. They should be widely read and remembered as the life record of one of the giants of his time.

